# Could Music Minimize Discomfort and Pain During Office-Based ENT Surgery?

**DOI:** 10.1155/2018/6480346

**Published:** 2018-11-14

**Authors:** Manuele Casale, Lorenzo Sabatino, Antonio Moffa, Giuseppe Oliveto, Vittorio Rinaldi, Andrea Costantino, Paola Vella, Andrea Ianni, Tommasangelo Petitti, Peter Baptista, Fabrizio Salvinelli

**Affiliations:** ^1^Associate Professor, Unit of Otolaryngology—Integrated Therapies in Otolaryngology, Campus Bio-Medico University, Rome, Italy; ^2^Unit of Otolaryngology—Integrated Therapies in Otolaryngology, Campus Bio-Medico University, Rome, Italy; ^3^Unit of Otolaryngology, University of Foggia, Foggia, Italy; ^4^Unit of Otolaryngology, Campus Bio-Medico University, Rome, Italy; ^5^Bio-Statistical Department, Campus Bio-Medico University, Rome, Italy; ^6^Unit of Otolaryngology, Clinica Universitaria of Navarra, Pamplona, Spain; ^7^Full Professor Unit of Otolaryngology, Campus Bio-Medico University, Rome, Italy

## Abstract

**Background:**

Video-assisted endoscopic radiofrequency inferior turbinate volume reduction (RFVTR) is one of the most common surgical therapies for inferior turbinate hypertrophy (ITH). Despite all the technical and surgical advancement, it is advisable to reduce as low as possible the intraoperative discomfort. The aim of this study is to evaluate the role of music in reducing patient discomfort during RFVTR.

**Materials and Methods:**

Twenty-three patients with chronic nasal obstruction due to ITH and candidate to RFVTR are included. Before the procedure each patient filled in a completed Italian version of the state anxiety questionnaire (State-Trait Anxiety Inventory), SNOT 22 questionnaire, VAS, and chose their favourite music to be played during RFVTR. All patients evaluate the intraoperative discomfort with a visual analog scale (VAS) and for each patient, vital parameters such as blood pressure and heart rate were recorded 15 minutes before the procedure, during and after RFVTR.

**Results:**

The intraoperative VAS scores during listening to music (5.7 ± 2.42 vs 6.7 ± 1.97; p< 0.05) were significantly lower, such as systolic BP (133.5 ±17.2 vs 136.78 ±16.8; p< 0.05) and heat rate (80.3 ±14.9 vs 81.7 ±15.5; p NS). During our survey, most of the patients preferred listening to classical music and none preferred rock music. No correlation was found between STAI 1-2 and intraoperative surgical discomfort evaluated both with VAS and cardiac parameters (systolic BP and HR).

**Conclusions:**

Music can be useful as a complementary method to control anxiety and reduce perception of pain in an office-based procedure, such as the RFVTR. The patient is more relaxed and experiences less discomfort; thus the surgeon and nurse can work with more confidence.

## 1. Background

Chronic nasal obstruction is a common symptom, and the most common cause of nasal obstruction in adults is inferior turbinate hypertrophy (ITH) [1]. It can be treated with medical therapy or, in patients refractory to medical management, it can be considered surgical procedures [2]. At least 13 mini-invasive different techniques have been introduced to reduce inferior turbinate volume but lack of consensus on the optimal technique. An ideal procedure for turbinate reduction should produce an improvement of nasal breathing with minimal discomfort or adverse reaction and should preserve the physiological function of the turbinates.

One of the most accepted surgical techniques is video-assisted endoscopic radiofrequency inferior turbinate volume reduction (RFVTR). It is easily bearable under topical anesthesia and, over the years, thinner instruments have been created to reduce the amount of discomfort and pain during the surgical procedure [1, 3].

Through targeted and controlled dosing it creates submucosal tissue shrinkage and contraction thus reducing turbinate volume. Because everything is happening submucosally, nothing should be changed with regard to the mucociliary transport system at the epithelial surface; one consequently maintains mucociliary function while achieving tissue volume reduction of the submucosa [4].

Several studies in the literature show that music can support patients during medical and surgical office procedure during which the patient is awake, with reducing pain, anxiety, and stress, focusing the patient's attention away from the negative stimulus to something desirable and relaxing and creating a positive environment during the procedure [5–9].

The primary aim of this study is to evaluate the role of music in reducing patient discomfort during RFVTR.

The secondary aims of this study are to evaluate the following:

- The correlation between preoperative anxiety trait and intraoperative discomfort
- The correlation between preoperative anxiety trait and the music benefit
- The correlation between preoperative nasal obstruction (Snot 22 and respiration VAS) and intraoperative discomfort.

## 2. Materials and Methods

Twenty-three consecutive patients (age between 24 and 75 years, mean age 47.3) affected by chronic nasal obstruction due to ITH and candidate to RFVTR were enrolled.

Patients affected by nasal polyposis, chronic rhinosinusitis, ongoing pregnancy, and nosebleeds, patients who already underwent nasal surgery, patients with marked septal deviation, patients immunocompromised, patients with bronchial asthma or chronic obstructive pulmonary disease, patients who had used antibiotics in the previous 30 days, and patients that chronically use immunosuppressive/corticosteroid were excluded.

All included patients underwent to RFVTR. To evaluate the role of music to alleviate pain and discomfort during the surgical procedure, a case controlled study was performed.

The music was administered only during the procedure on one nostril, the contralateral one was used as control. Side and order of the music administration was randomly selected. The time interval between the two nostrils procedures (one with music, the other without music) was at least 5 minutes.

The study, as defined in the Helsinki Convention, was approved by the Ethics Committee of the University Campus Bio-Medico in Rome (Protocol: 31.15 TS ComEt CBM) and every patient gave his informed consent.

Each patient was subjected to the following procedures.

### 2.1. Preoperatively

- History and pre- and postoperative nasal endoscopic;
- Subjective nasal respiratory evaluation of each nostril through preoperative 10-score visual analog scale (VAS);
- Subjective STAI form Y-1 and Y-2 preoperative questionnaire,
- Subjective SNOT-22 questionnaire
- Evaluation of heart rate and blood pressure

### 2.2. Intraoperatively

- Choice of favourite musical genre
- Evaluation of pain through 10-score visual analog scale (VAS), heart rate, and blood pressure during RFVTR in the nostril with music and in the contralateral one without music

### 2.3. Postoperatively

- Evaluation of heart rate and blood pressure

### 2.4. Visual Analog Scale

The VAS consists of a graduated scale with values from 0 to 10, whereas 0 corresponds to no symptoms and 10 corresponds to a feeling of maximum symptomatology. The VAS on nasal breathing is directly administered to the patient, who has to assess his nasal obstruction considering one nostril at a time; so the patient is asked to close one of its two nostrils with his finger before giving the score to the airflow through the contralateral free nostril.

The VAS on pain of the surgical procedure was calculated asking to the patient during the tissue reduction of the tail of each inferior turbinate, to minimize operator bias and variation of the shape of the nostrils [10].

### 2.5. Nasal Endoscopy

Nasal endoscopy with flexible nasal fiber endoscope is carried out to elicit information relevant to inclusion criteria and to evaluate the ITH grade. All included patient candidates to RFVTR showed severe or obstructive inferior turbinate hypertrophy and the nasal septum substantially in axis.

### 2.6. STAI Questionnaire

The STAI (State Trait Anxiety Index) for adults was used to assess anxiety. It consists of 40 self-reported items that measure state (STAI form Y-1) and trait (STAI form Y-2) anxiety. Scores range from 20 to 80, and the lower the score the lower the anxiety level. In particular, the STAI Y- 1 is designed to assess momentary or situational anxiety. The STAI Y-2 is designed to assess trait anxiety, with questions that explore how the subject feels habitually. STAI Y-1 was administrated twice, before and at the end of the procedure, whereas STAI Y-2 was administered only before the procedure to assess homogeneity between trait anxieties of both groups [11].

### 2.7. Snot-22

It consists of 22 questions about of symptoms and social/emotional consequences of rhinosinusitis.

The patient is asked to complete the questionnaire about his problems, as they have been over the past two weeks. Considering how severe the problem is and how frequently it happens, the patient has to circle the number; these are ranging from 1 to 5; the higher the number, the greater the frequency and severity of that particular symptom [12].

### 2.8. Music Administration

Before the start of the procedure, patients were asked to indicate their favourite music to be played during RFVTR; they could choose among selected pop, jazz, classical, or rock or indifferent. Music was administered through a stereo to a volume of 50 to 60dB [13] during RFVTR of one nostril, while the surgery was performed on the other nostril without music.

### 2.9. Heart Rate and Blood Pressure

Those basical parameters were usually measured by the nurse during RFTVR. Their change basically reflects the amount of anxiety or pain of the patients [6].

### 2.10. Surgical Procedure

The patients were subjected to RFVTR; we followed the methods of Casale M. et al. [4].

To deliver this energy we used the Somnoplasty System (Somnus Medical Technologies, Inc., Bartlett, TN, USA) and the SP 1100 turbinate handpiece (40 mm-long needle electrode consisting of a distal 15-mm active portion and a proximal 25 mm insulated part) with thermocouples within the electrode to allow the surgeon feedback during treatment: this included tissue temperature, power used, and total energy delivered. Before the surgical procedure, a cotton pledget soaked with mepivacaine and adrenaline solution was introduced into each inferior meatus. The patient was placed in the supine position and after 10 minutes, under 0° and 30° endoscopic vision, the active 10 mm portion of the electrode and at least 2 mm of the insulation were inserted submucosally while the energy was delivered into three different sites of each turbinate (anterior, middle, and posterior portion). The energy delivered for each puncture was 300 J with an average duration of 59 ± 16 seconds and a plateau tissue temperature of 75 ±0.6°C. After treatment, all patients were discharged without any limitation of normal daily activities and no nasal pack was needed.

### 2.11. Statistical Analysis

Stata (StataCorp., Texas, USA) and Excel (Microsoft Store, Washington, USA) has been used for statistical analysis.

## 3. Results

The mean preoperative values of SNOT 22 were 36,47 (±24) with a preoperative mean Subjective nasal respiratory VAS of 6.7 (±1.3); the STAI mean values were 44.87 (±4.6) and 45.96 (±5.2) for STAI I and II, respectively. At the nasal endoscopy, all patients showed grade 3 ITH.

Analyzing vital parameters, our patients showed mean preoperative systolic blood pressure (BP) 130.3 (±18) with mean HR 76.2 (±14.8) and intraoperative mean systolic BP during RFVTR without music (RFVTRwm) of 136.78 (±16.8) with an HR of 81.7 (±15.5) while during music (RFVTRm) a BP of 133.5 (±17.2) with a HR 80.3 (±14.9).

**Figure 1:**
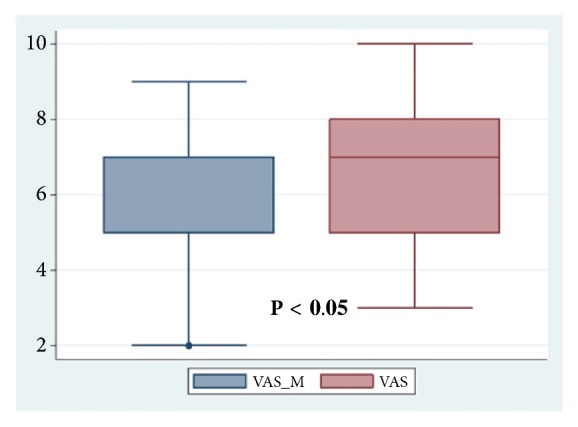
VAS of pain.

Comparing the VAS scores with and without music, the VAS during listening to music was significantly lower (RFVTRm 5.7 ± 2.42 vs RFVTRwm vs 6.7 ± 1.97; p<0.05) as shown in Figure 1.

**Figure 2:**
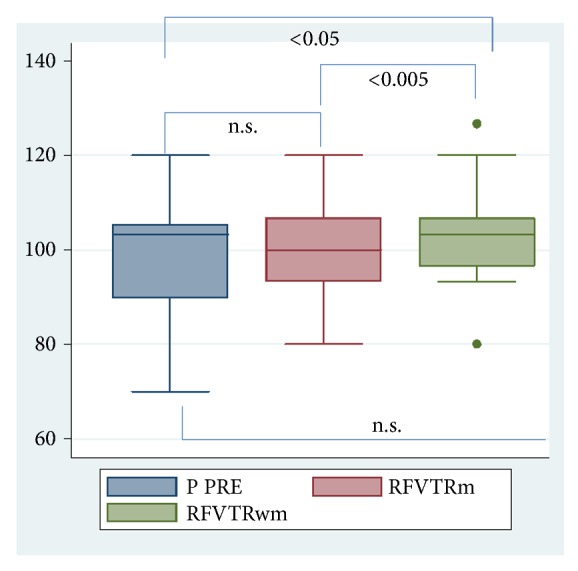
VAS of systolic pressure.

The significant VAS reduction in RFVTRm was confirmed by a statistically significant lower systolic BP compared to RFVTRwm; in particular, the intraoperative systolic BP significantly increase without music (P<0.05), while only a slight difference during listening to music was found (P=NS), as shown in Figure 2. Also HR was lower during listening to music, even if the difference was not statistically significant.

There is no significant statistical correlation between preoperative respiration VAS, snot 22 and STAI 1-2, and intraoperative surgical discomfort. During our survey, 50% of patients preferred listening to classical music, 35% preferred pop music, 25% preferred jazz, and none preferred rock music.

## 4. Discussion

RFVTR uses radiofrequency heating to induce submucosal tissue destruction. The aim of RFVTR is obtain an improvement of the nasal breathing the physiological function of the turbinate whit minimal discomfort or adverse effects [4].

However, it has been well known that any surgical procedures, is associated with increased anxiety and that this emotional state can lead to both psychologic and physiologic responses.

To alleviate this intraoperative discomfort the potential role of music in some surgical procedures were well documented; in particular, music relieved anxiety and improved tolerance for endoscopic examination in patients undergoing colonoscopy was also useful during hysteroscopy and may have a role on promoting regain of consciousness [7, 14–16].

To our knowledge, the present study is the first case control study to investigate the effects of music on pain perception during office-based rhinologic surgical procedures with awake patient.

There are various theories and evidences about how music can help during surgical acts. It is well known that music could stimulate brain, acting on specific areas involved in processes of memory, learning, and multiple motivational and emotional states [17].

Our playlists for classical, pop, jazz, and rock music have been structured to include music as relaxing as possible. It is important to note that no patient chose the rock playlist, indicating a natural tendency to associate smooth music with a relaxing effect; indeed, the type of music most often used in health care settings was smooth music. However most of our patients chose classical music may be because it is usually considered more relaxing, which may be why so many patients chose this playlist.

Our results show that patients during listening to music show less discomfort during the surgical procedure as confirmed by positively affecting the activity of the sympathetic nervous system [18], as shown by the significantly lower systolic blood pressure and HR during listening to music in comparison to procedure without music. Our study did not show any correlation between pain and anxiety, although in the literature such evidence was often reported [19–21].

A time interval of 5 minutes between with and without music may not be long enough and this may be consider a limit of our study: however we could not procrastinate this time interval because we considered not kind to the patient to prolong the stay on the operating table and because of organizational reasons of the operating theatre.

The validity of this study is also limited by the lack of the ability to blind the patient; considering that the perception of pain is highly subjective, we have preferred comparing two groups and evaluating the music benefit in the same patient. Therefore the difference of discomfort VAS between music and no-music, although statistically significant, may not bring a real clinical significance.

Music may promote relaxation and decrease anxiety, resulting in a higher threshold for pain [15, 16]. Alternatively, music may distract from painful sensation and thereby reduce anxiety responses. The distraction may also work at the cognitive level; patients are distracted from their worries and anxiety-provoking thoughts and instead focus on more pleasant stimuli, thereby reducing their level of tension, which in turn could predictably have a beneficial effect on reducing pain and distress. We are sure that the beneficial effect of music on patient reflects positively also on the surgical équipe that is more confident and focused on surgical procedure.

Our data show that music can have a role as complementary treatment to reduce anxiety and minimize discomfort during ENT office-based procedures, such as inferior turbinate volume reduction. On the basis of recent literature and our preliminary data we encourage to listen to music in all ENT office surgical procedures and to investigate the potential advantage of music listening also in other surgical fields.

## Data Availability

The scientific data used to support the findings of this study are included within the article.
